# The Formation of Uric Acid and Its Effects on the System

**Published:** 1894-03-31

**Authors:** Alexander Haig

**Affiliations:** Physician to the Metropolitan Hospital and to the Royal Hospital for Children and Women


### Medical Progress and Hospital Clinics.

[*The Editor will be glad to receive offers of co-operation and contributions from members of the profession. All letters should be addressed to* The Editor, The Lodge, Porchester Square, London, W.]

### I.—THE FORMATION OF URIC ACID AND ITS EFFECTS ON THE SYSTEM.

By Alexander Haig, M.A. and D.M. Oxon., F.R.C.P., Physician to the Metropolitan Hospital and to the Royal Hospital for Children and Women.

That one word "formation" still sums up for many the origin of all troubles connected with uric acid, but this is far from being my belief or the result to which my researches seem to point, as the facts I shall now shortly mention may serve to show. In 1,284 days (carrying my observations, on myself up to some eight months ago) I excreted 404,793 grains of urea, 14,353 grains of uric acid, and 65,386 grains of acid, reckoned as oxalic acid. This gives a relation of uric acid to urea of 1 to 28 and a relation of acid (acidity) to urea of 1 to 6·1, and the figures divided by the number of days give 315 grains of urea, 11·1 grains of uric acid, and 50·9 grains of acid per day. Now this 11·1 grains of uric acid represents the amount of that substance I formed in a day, plus such amount as was daily introduced direct into the body ready formed, and plus such quantity as was already in my body at the beginning of the experiment and has been since excreted.

It must suffice to say here that I discovered by experiment on myself and others that uric acid urates, caffeine hypoxanthin or other members of the xanthin group when taken by mouth increase proportionately with the dose taken the amount of uric acid in the body and blood, and the amount passing in the urine.*

Further, I already knew that meat, fish, fowl, and game all contained a small but definite percentage of uric acid, or its congeners the xanthins, and it thus became possible to calculate approximately the amount of uric acid I had directly introduced into my body in these articles of food over the period named. (For further details on these matters see my paper in the *Journal of Physiology*, Vol. XV., p. 167, and "Uric Acid," London, J. and A. Churchill, second edition, now in the press.)

I considered that at the beginning of the 1,284 days I had stored in my body some 300 grains, which were excreted but not formed during this period, and I took during the same period 123 grains of uric acid and xanthin, &c. (for experimental purposes); subtracting these 423 grains from the total uric acid, we get the daily excretion reduced to 10·8 grains, and if we put the daily introduction in animal food at 1·8 grains for the sake of getting whole numbers, we get, by subtracting this, my probable daily formation of uric acid at 9·0 grains, having a relation to the 315 grains of excreted urea of 1 to 35. Our problem then works out as follows: Daily formation of uric acid in body, 9·0 grains; daily introduction of uric acid into body, 1·8 grains; daily excretion from stores previously in body, ·23 grains; daily introduction of uric acid by experiment, ·09 grains; excretion total equals 11·12 grains. If we disregard decimals, it may be said that of eleven grains of uric acid which I daily excreted, nine grains were formed in my body out of the nitrogen, which also furnished the 315 grains of urea, and two grains were introduced in the food as ready-formed uric acid.

* By a similar experiment on fowls, W. von Mach has found that the administration of hypoxanthin increases the excretion of uric acid. Archiv. für Exp. Pathol. u. Pharmak. Bd. 23, S. 148.

I have never seen anything in my investigation of my own excreta or those of others which would lead me to think that either I or they ever form uric acid in greater relative proportion to urea than somewhere about 1 to 35, and I further see cause to believe that the great accumulations of urates found in the liver, spleen, joints, cartilages, and urinary passages are all due and can easily be accounted for by failure of excretion, which failure I can myself produce at any time with the greatest of ease. But while I put my own introduction of uric acid at from 1·8—2·0 grains a day, I believe that in the case of those who eat largely of animal food nearly as much as this may be introduced with each of their three chief meals.

It is little wonder, then, that in Persia, where only the rich eat meat, gout is known as the rich man's disease (see "Proceedings of the Royal Medical and Chirurgical Society," Third Series, Vol. v., p. 46, where this interesting fact is contributed to a discussion on one of my papers by the Physician to the Shah), or that the children of the rich should be specially prone to suffer from anæmia (see further on, and also *British Medical Journal*, September, 1893).

If this is the case the uric acid problem is much more simple than has hitherto been supposed, for in place of an unknown and unlimited formation, we have a formation which can be approximately calculated in one moment from the urea in the proportion 1 to 35; and an introduction, which can also be approximately calculated from the quantities of uric acid, xanthin, &c., found in the various foods used, or from urea in the proportion of 1 to 175. And if anyone comes to us suffering from an excess of uric acid, we can tell him not only how he came to have this excess, but also how he can get rid of it, and prevent its reaccumulation.

This brings us to the effects of uric acid on the system, and the signs by which its excess may be recognised. But here again I must confine myself to a mere outline of the facts, referring my readers for further details to the book above mentioned. The simplest effect of urates in the body is the irritation they produce in the tissues, when present in them in considerable quantity, while they are in solution or in process of crystallization; and we know, on the other hand, that when they have been deposited they may remain quiet for a long time, often without causing any irritation. Such effects are seen in acute rheumatism, where they may act only for a few hours, or in gout, where they act for a longer period, and may eventually be deposited in mass; and both these forms of irritation by urates have these important characteristics in common—that substances which diminish the solubility of urates in the blood, such as acids, iron, lead, mercury, copper, silver, and other metals make them worse, while substances which increase the solubility of uric acid in the blood, such as alkalies and the salicylates, make them better. Thus the common cause of acute rheumatism is a general fall in the alkalinity of the blood, and of acute gout a more or less local fall in the alkalinity of the blood and tissue fluids, and such alterations of alkalinity can often be produced almost at pleasure, and Nature's modes of action imitated to almost any required extent.

The other and by far the more important and extensive action of urates in the system is the contraction or obstruction which they bring about in the small vessels throughout the body, which results, on the one hand, in a slow and deficient circulation in all the capillaries in the body, and, on the other, in high blood pressure in the arteries on the proximal side of the obstruction, the absolute amount of this pressure depending on the amount of obstruction in front, and the power of the heart to keep up the pressure behind.

There is no space to state here the facts that have led me to conclude that uric acid does cause this obstruction to the circulation in the minute vessels, but it can quite easily be demonstrated that the amount of contraction or obstruction in the vessels of the kidney which regulates the amount of water it excretes is strictly proportional to the amount of uric acid which is passing through this organ from the blood to the urine, and by increasing or diminishing the amount of urate which thus passes, as it is quite easy to do with drugs, the corresponding excretion of water can be controlled. But such changes in their circulation must obviously affect the function, nutrition, and eventually the structure of all the organs and tissues of the body, and I can only mention a few of the more important here. Thus there is considerable evidence to show that the structure of the blood is affected either directly by the presence of an excess of uric acid, or indirectly by the effects of this substance on the circulation of such important metabolic tissues as the liver and spleen, the muscles, or the skin; and the changes thus brought about result in acute conditions in paroxysmal hæmoglobinuria or albuminuria, and in more chronic conditions in anæmia and perhaps Bright's disease. (See "Med. Society Trans.," Vol. XV., p. 143; "St. Bartholomew's Hosp. Reports," Vol. XXVIII., p. 29; and *British Medical Journal*, September, 1893.) Then the high blood pressure will obviously affect the vessels producing strain, degeneration, deficient nutrition, and atheroma, and the heart producing hypertrophy and valvular strain, followed by degeneration and dilatation. Further, the arterial tension affects in an important manner, as I have pointed out, the intra-cranial circulation bringing about stasis and hyperæmia, which may possibly account for headache, mental depression, fits, vertigo, and other minor functional disturbances, as hysteria. (See *Brain*, Spring and Summer number, 1893.)

In a similar way the high blood pressure or obstructed capillaries will affect the bronchial and pulmonary circulation in the thorax, accounting for the scanty excretion of water from the lungs, which corresponds with a scanty excretion of water in the urine, and probably having a good deal to do with the causation of chronic bronchitis and of asthma. (See "International Clinics," Vol. III., third series and following number; and "Medico-Chirurgical Transactions," Vol. LXXVI., p. 133.) Then, when you get a similar deficient circulation in the digestive organs, you get deficient secretion from the stomach, the liver, the pancreas, and the salivary glands, from both of which (deficient circulation and deficient secretion) result deficient digestion and absorption of food, with a certain amount of fermentation and putrefaction, formation of wind and other abnormal products, resulting in pain and discomfort. A scanty secretion of urine is the constant result of a similar deficient circulation in the kidneys; and so much is this the case that the urinary water actually varies from hour to hour and day to day inversely as the uric acid; the greater the excretion of uric acid the less the excretion of water, and *vice versa*, and this fact seems to me to be the strongest possible proof that uric acid acts on the arterioles in the way I have pointed out, and, indeed, the excretion of water from the lungs and all the digestive glands is similarly related to the excretion of uric acid in the urine, because this is an index of the amount passing through the blood, on which depends the capillary circulation of the whole body.

Similarly in the skin we get all kinds of alteration in function, nutrition and structure from mere harshness and dryness to the atrophy of Bright's disease, and from temporary coldness and pallour to the structural changes of Raynaud's disease, all similarly related to the excretion of urates and uricacidæmia. Such, I believe, are some of the more important troubles which are due to an excess of uric acid in the blood, and I shall deal in a future paper with the best methods of prevention and cure.

